# 柱前衍生-超高效液相色谱-串联质谱法检测茶叶中草铵膦及其代谢物残留量

**DOI:** 10.3724/SP.J.1123.2025.09022

**Published:** 2026-09-08

**Authors:** Ruifeng BI, Shihui FU, Suocheng DENG, Meng FU, Weiwei ZHANG, Yuhua SONG

**Affiliations:** 梅里埃检测技术（青岛）有限公司，山东 青岛 266111; Mérieux NutriSciences Testing Technology （Qingdao） Co. ，Ltd. ，Qingdao 266111，China

**Keywords:** 柱前衍生, 超高效液相色谱-串联质谱, 草铵膦, *N*-乙酰基草铵膦, 3-（甲基膦基）丙酸, 原乙酸三乙酯, 茶叶, precolumn derivatization, ultra-high performance liquid chromatography-tandem mass spectrometry （UHPLC-MS/MS）, glufosinate, *N*-acetyl-glufosinate, 3-［hydroxy（methyl）phosphinoyl］propionic acid, triethyl orthoacetate, tea

## Abstract

建立了超高效液相色谱-串联质谱（UHPLC-MS/MS）检测茶叶中草铵膦（GLU）及其代谢物*N*-乙酰基草铵膦（NAG）和3-（甲基膦基）丙酸（MPP）的高灵敏度分析方法。样品采用超纯水提取，提取溶液经冷冻、高速离心后，取上清液氮吹至干，以乙酸为催化剂，分析物与原乙酸三乙酯进行柱前衍生。NAG生成与GLU相同的反应产物，因此与GLU合并检测。选择Agilent ZORBAX Eclipse Plus C18色谱柱（100 mm×2.1 mm， 1.8 μm）作为固定相，以0.1%甲酸水溶液（含5 mmol/L甲酸铵）和乙腈为流动相进行梯度洗脱，在电喷雾正离子模式下进行检测，多反应监测模式采集数据，同位素内标法定量。结果表明，GLU/NAG和MPP在0.5~20 µg/L范围内均有良好的线性关系（*r*^2^>0.995），检出限为2.2~5.3 µg/kg，定量限为25 µg/kg；取绿茶和红茶空白样品进行0.025、0.05、0.1和0.5 mg/kg 4个水平的添加回收试验，目标化合物的平均回收率为83.9%~105.0%，日内精密度（*n*=5）为1.9%~6.9%，日间精密度（*n*=10）为2.6%~10.3%。采用该方法检测34批次茶叶样品，样品的阳性检出率为26.5%。总之，该方法简单、可靠、实用，适合各种茶叶中草铵膦及其代谢物残留量的检测。

## Abstract (en)

Tea is an important cash crop in China， and pesticide residues in tea products are among the main issues affecting tea exports. Glufosinate （GLU） is a widely used herbicide in tea gardens and its residues in tea samples have raised health concerns. However， there are few reports on analytical methods for the simultaneous determination of GLU and its metabolite residues in tea. Therefore， the development of suitable analytical methods to monitor the content of GLU and its metabolite residues in tea is essential to ensure compliance with the export quality standards of tea products. In this study， a sensitive analytical method was developed for the determination of GLU and its two main metabolites， *N-*acetyl-glufosinate （NAG） and 3-［hydroxy（methyl）phosphinoyl］propionic acid （MPP）， in tea. The method is based on ultra-high performance liquid chromatography-tandem mass spectrometry （UHPLC-MS/MS） coupled with precolumn derivatization. Tea samples （2.00 g） were extracted with 20 mL of ultrapure water by shaking， and followed by centrifugation. After freezing treatment， aliquots of the extract were centrifuged at 15 000 r/min. Then 200 μL of the supernatant was evaporated to complete dryness using nitrogen blowdown. The resulting residue was derivatized with triethyl orthoacetate （TEOA） at 80 ℃ for 1.5 h， in the presence of acetic acid as the catalyst. The derivatization mixture was evaporated to dryness and reconstituted with methanol. NAG was transformed into the same reaction product as GLU， and therefore quantified together with GLU. The derivatization products were separated on an Agilent ZORBAX Eclipse Plus C18 column （100 mm×2.1 mm， 1.8 μm）. The mobile phase consisted of an aqueous solution of 0.1% （*V/V*） formic acid （containing 5 mmol/L ammonium formate） as phase A and acetonitrile as phase B. The gradient elution was carried out as follows： 0–0.5 min， 90%A； 0.5–7.5 min， 90%A–80%A； 7.5–8.0 min， 80%A–10%A； 8.0–10.0 min， 10%A； 10.0–10.1 min， 10%A–90%A； 10.1–12.0 min， 90%A. The separated compounds were detected by tandem mass spectrometry with positive electrospray ionization （ESI^+^） in multiple reaction monitoring （MRM） mode， and stable isotope-labeled internal standards were used for quantitative analysis. Good linearity was obtained in the 0.5–20 μg/L range； the correlation coefficients （*r*^2^） were higher than 0.995， the limits of detection were 2.2–5.3 µg/kg and the limits of quantification were 25 µg/kg. For two representative matrices of green tea and black tea， the average recoveries of GLU and MPP spiked at four levels of 0.025， 0.05， 0.1， and 0.5 mg/kg were 83.9%–105.0%. The intra-day precisions （*n*=5） ranged from 1.9% to 6.9% and the inter-day precisions （*n*=10） ranged from 2.6% to 10.3%. Furthermore， the developed method was used to detect GLU and its metabolite residues in 34 tea samples， and the positive detection ratio was 26.5%. In summary， the proposed method is simple， reliable， practical， and suitable for determining GLU and its metabolite residues in various types of tea.

茶叶是我国重要的经济作物。2024年，我国茶叶总出口量37.41万吨，占全球茶叶供应量的40%。尽管茶叶产业发展迅速，农药残留问题却依然突出，直接影响了茶叶产业的健康发展^［1］^。草铵膦（glufosinate，GLU）是赫斯特公司于20世纪80年代开发的一种非选择性除草剂，目前使用量仅次于草甘膦，是全球第二大除草剂，其作用机制是抑制谷氨酰胺合成酶的活性，导致谷氨酰胺合成受阻，氮代谢紊乱，造成铵的过量积累，从而干扰植物的代谢，使植物死亡^［2］^。草铵膦具有起效快、在土壤中降解迅速、残留水平低的特点^［3］^，在茶园中被广泛使用。

随着草铵膦使用量的增加，其残留问题与潜在风险也日益引发公众关切，世界各个国家和地区对茶叶中的草铵膦残留制定了严格的规定^［4］^。欧盟、英国、摩洛哥进口茶叶执行0.1 mg/kg的限量标准；日本肯定列表制度规定最大残留限量为0.3 mg/kg，中国食品安全国家标准GB 2763-2026规定最大残留限量为0.5 mg/kg。关于草铵膦的残留物定义，除GB 2763-2026规定为草铵膦母体外，国际上普遍定义为草铵膦、3-（甲基膦基）丙酸（3-［hydroxy（methyl）phosphinoyl］propionic acid，MPP）和*N*-乙酰基草铵膦（*N*-acetyl-glufosinate，NAG）之和，以草铵膦表示；不过欧盟认为，对于非转基因作物而言，残留物定义为“草铵膦与3-（甲基膦基）丙酸之和，以草铵膦表示”也可以接受^［5］^。总之，建立茶叶中草铵膦及代谢物的检测方法非常必要。

草铵膦及其代谢物的检测方法可分为衍生法和非衍生法。衍生法包括9-芴甲基氯甲酸酯（FMOC-Cl）衍生法^［6-10］^、原乙酸三甲酯衍生法^［11-13］^、重氮甲烷衍生法^［14］^、*N*-（叔丁基二甲基硅烷基）-*N*-甲基三氟乙酰胺衍生法^［15］^等；其中FMOC-Cl衍生法应用最多，因MPP没有能与FMOC-Cl反应的活性基团，FMOC-Cl衍生法不适用于MPP的检测。农药残留专家联席会议报告中关于草铵膦代谢物的研究主要采用原乙酸三甲酯衍生法^［16］^。重氮甲烷可以同时和羟基、羧基和氨基进行反应，但潜在爆炸性和致癌性限制了其在实验室中的广泛使用。近年来，随着极性化合物专用色谱柱技术的发展，非衍生化检测方法的报道日益增多^［17-27］^；然而，对于茶叶等复杂基质，非衍生化法面临灵敏度低和基质干扰等挑战。

本研究采用原乙酸三乙酯（triethyl orthoacetate，TEOA）对目标化合物进行衍生化处理，并优化了衍生化条件、质谱参数和色谱条件，建立了茶叶中草铵膦及其代谢物的检测方法。该方法前处理简单，灵敏度高，稳定性好，为研究茶叶中草铵膦残留及保障茶叶出口提供了技术支持。

## 1 实验部分

### 1.1 仪器与试剂

Agilent 1290B Infinity/6495C超高效液相色谱-三重四极杆质谱仪（美国Agilent公司）；Geno/Grinder型组织研磨机（美国SPEX SamplePrep公司）；Vortex Genie 2型旋涡混合器（美国Scientific Industries公司）；MTN-2800W型氮吹仪（天津奥特赛恩斯仪器有限公司）；X1R型台式离心机（美国Thermo-Fisher公司）；D3024型高速微量离心机（大龙兴创实验仪器（北京）有限公司）；DKZ系列电热恒温振荡水槽（上海一恒科技有限公司）；KQ-300ES型超声波清洗仪（昆山市超声仪器有限公司）。

GLU（纯度98.0%，CAS号：77182-82-2，北京坛墨质检科技有限公司）；GLU-D_3_（纯度99.4%，CAS号：1323254-05-2，德国Dr. Ehrenstorfer公司）；NAG（99.5 μg/mL，CAS号：73634-73-8，北京曼哈格生物科技有限公司）；MPP（纯度99.9%，CAS号：15090-23-0）、MPP-D_3_（100 μg/mL、CAS号：66992-42-5），天津阿尔塔科技有限公司。甲醇、乙腈、甲酸铵（色谱纯，上海安谱实验科技股份有限公司）；甲酸和乙酸（色谱纯，美国Merck公司）；原乙酸三乙酯、原乙酸三甲酯、原甲酸三乙酯（纯度≥97%，上海阿拉丁生化科技股份有限公司）；HLB 固相萃取柱（50 mg/3 mL，天津博纳艾杰尔科技有限公司）；多壁碳纳米管（multi-walled carbon nanotubes，MWCNTs）（直径10~20 nm，长度10~30 μm，江苏先丰纳米材料科技有限公司）；实验用水为超纯水（18.0 MΩ·cm，产自Milli-Q纯水仪）；实际茶叶样品购自本地超市。

### 1.2 标准溶液的配制

对于固体标准品，准确称取10.0 mg标准品，用超纯水溶解并配制成质量浓度为1 000 mg/L的标准储备液。标准工作溶液：准确吸取上述标准储备液和液体标准品适量，用水稀释配成质量浓度分别为0.05、0.5和5 mg/L的标准工作液。内标标准工作溶液：准确吸取各氘代同位素内标标准溶液适量，用乙腈稀释配成质量浓度分别为0.05和0.5 mg/L的内标标准工作液。以上溶液于４ ℃冰箱中避光保存。

分别移取10、20、40、100 μL质量浓度为0.05 mg/L的标准工作液，20、40 μL质量浓度为0.5 mg/L的标准工作液至15 mL塑料离心管中，各加入20 μL质量浓度为0.05 mg/L的内标标准工作液，氮吹，衍生，再次氮吹，1 mL甲醇复溶，制备得到目标物质量浓度依次为0.5、1、2、5、10和20 μg/L，内标质量浓度为1 μg/L的系列校正标准工作液。

### 1.3 实验方法

#### 1.3.1 样品前处理

称取2.00 g样品至50 mL离心管中，添加200 μL 0.5 mg/L的内标溶液，加入20 mL超纯水，涡旋使样品浸润，静置30 min，以1 000 r/min转速振荡提取1 min，然后以7 000 r/min转速离心5 min；移取1 mL上清液至2 mL离心管中，于‒20 ℃冰箱冷冻10 min，取出后以15 000 r/min高速离心5 min。移取200 μL上清液至15 mL离心管中，于45 ℃水浴氮吹至完全干。加入0.5 mL乙酸和2 mL原乙酸三乙酯，水浴超声2 min，涡旋10 s，于80 ℃水浴反应1.5 h，然后在45 ℃水浴中氮吹至干；加入1 mL甲醇，超声复溶，过0.22 μm尼龙滤膜，上机分析。

#### 1.3.2 色谱-质谱条件

色谱柱：Agilent Eclipse Plus C18柱（100 mm×2.1 mm，1.8 μm）；流动相A为0.1%甲酸水溶液（含5 mmol/L甲酸铵），流动相B为乙腈。梯度洗脱程序：0~0.5 min，90%A；0.5~7.5 min，90%A~80%A；7.5~8.0 min，80%A~10%A；8.0~10.0 min，10%A；10.0~10.1 min，10%A~90%A；10.1~12.0 min，90%A。流速为0.3 mL/min，柱温为40 ℃，进样量为1 μL。

在电喷雾正离子模式及多反应监测（multiple reaction monitoring，MRM）模式下采集数据。毛细管电压，3 000 V；干燥气温度，250 ℃；干燥气流速，14 L/min；雾化气压力，241.3 kPa（35 psi）；鞘气温度，350 ℃；鞘气流速，11 L/min。目标化合物的保留时间和质谱参数见表1。

**表1:** 目标化合物及其同位素内标的保留时间和质谱参数

Compound	*t*_R_/ min	Molecular formula	*M*_r_	Precursor ion （*m/z*）	Product ions （*m/z*）	Collision energies/eV
GLU/NAG-TEOA	4.31	C_11_H_22_NO_5_P	279.12	280.1	164.0^*^， 238.0， 206.0	25， 13， 13
MPP-TEOA	6.03	C_8_H_17_O_4_P	208.09	209.1	79.1^*^， 135.1， 163.0	35， 15， 8
GLU-D_3_-TEOA	4.31	C_11_H_19_D_3_NO_5_P	282.14	283.1	167.1	20
MPP-D_3_-TEOA	6.00	C_8_H_14_D_3_O_4_P	211.11	212.1	138.0	16

GLU： glufosinate； NAG： *N*-acetyl-glufosinate； TEOA： triethyl orthoacetate； MPP： 3-［hydroxy（methyl）phosphinoyl］propionic acid； * quantitative ion.

## 2 结果与讨论

### 2.1 质谱方法优化

移取GLU单标溶液与原乙酸三乙酯在80 ℃水浴反应，得到衍生产物溶液。对GLU衍生产物溶液进行一级质谱扫描，在正离子模式下得到准分子离子峰*m/z* 280.1；以*m/z* 280.1的离子为母离子进行二级质谱离子扫描，并对碰撞能量等参数进行优化，得到GLU衍生产物的产物离子和对应的最优碰撞能量。采用相同方法衍生NAG、MPP、GLU-D_3_和MPP-D_3_，并对各衍生产物进行一级和二级质谱扫描，获得各衍生产物的质谱参数。

根据理论和实验结果推测GLU、NAG、MPP及内标的衍生反应机理见图1，分子中的磷羟基和羧基发生了乙酯化反应，氨基发生了乙酰化反应；其中，GLU和NAG衍生后生成的产物相同，统一表示为GLU/NAG-TEOA。

**图1:**
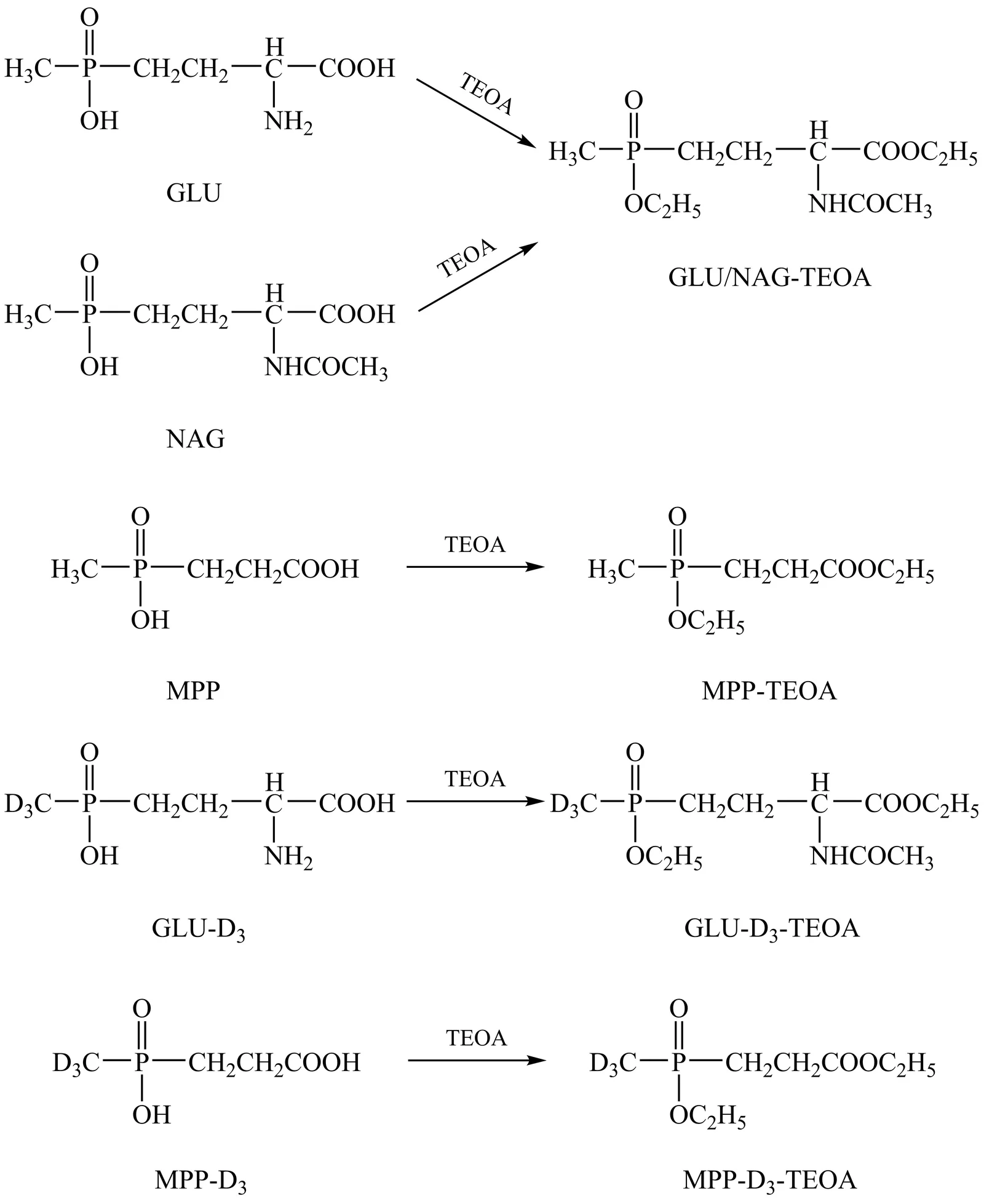
GLU、NAG、MPP、GLU-D_3_、MPP-D_3_与原乙酸三乙酯的衍生反应

### 2.2 色谱条件优化

#### 2.2.1 色谱柱选择

草铵膦及其代谢物的衍生化反应封闭了分子中的羟基、羧基和氨基极性基团，使分子极性大大降低。本实验以乙腈-0.1%甲酸水溶液（含5 mmol/L甲酸铵）作为流动相，比较了Agilent ZORBAX Eclipse Plus C18 （100 mm×2.1 mm，1.8 μm）、Acquity UPLC HSS T3 （100 mm×2.1 mm， 1.8 μm）和Capcell Pak ADME HR （100 mm×2.1 mm， 2 μm） 3种不同色谱柱对目标化合物的分离效果。结果显示，采用3种不同色谱柱均能获得良好的分离效果和质谱响应，区别仅是化合物的保留时间不同，其中，化合物在ADME色谱柱上的出峰时间最长，T3色谱柱次之，在Eclipse色谱柱出峰时间最短。由此可见，不同色谱柱均适用于草铵膦及代谢物衍生产物的色谱分离。由于Eclipse色谱柱相对于其他两种色谱柱使用成本更低，且在农残检测中通用性更强，因此，本实验选择Agilent ZORBAX Eclipse Plus C18（100 mm×2.1 mm，1.8 μm）色谱柱进行后续实验。

#### 2.2.2 流动相的选择

在LC-MS/MS分析中，流动相组成不仅影响分析物在固定相上的色谱保留行为，同时影响质谱的检测灵敏度。本实验对比了甲醇-0.1%甲酸水溶液，甲醇-0.1%甲酸水溶液（含5 mmol/L甲酸铵）、乙腈-0.1%甲酸水溶液，乙腈-0.1%甲酸水溶液（含5 mmol/L甲酸铵）4种流动相。结果显示，当采用乙腈-0.1%甲酸水溶液（含5 mmol/L甲酸铵）作为流动相时，GLU、MPP及对应内标具有更好的峰形和响应，且通过优化梯度洗脱程序，目标化合物和附近干扰峰能够实现基线分离。绿茶加标样品中GLU、MPP及同位素内标的MRM色谱图见图2。由于加标样品中的NAG和GLU经衍生后均变为GLU/NAG-TEOA，在色谱图上无法区分，因此不能同时加标GLU和NAG，图2仅展示了单独加标GLU的色谱图。

**图2:**
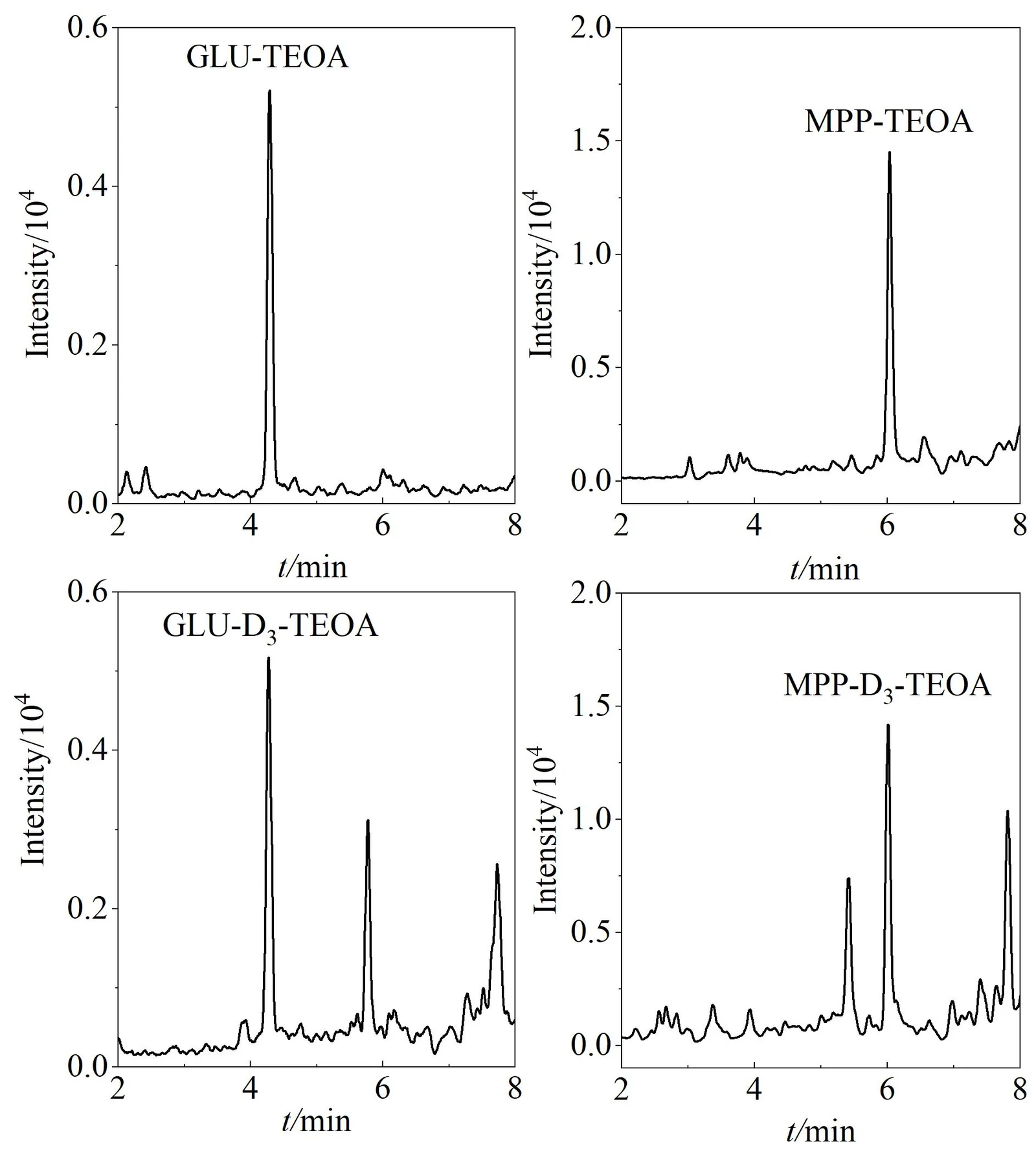
绿茶加标样品（0.05 mg/kg）中GLU、MPP及其同位素内标衍生产物的MRM色谱图

### 2.3 衍生方法的建立

#### 2.3.1 衍生试剂的选择

本研究使用原乙酸三甲酯、原乙酸三乙酯和原甲酸三乙酯3种原酸酯试剂作为衍生化试剂，并进行了比较。以原乙酸三甲酯为衍生试剂，原乙酸三甲酯与目标分析物的衍生反应机理见文献［^5^］。结果显示，衍生产物在C18反相色谱柱上保留弱，出峰早，基质干扰峰多。以原乙酸三乙酯为衍生试剂，分析物中羧基和磷羟基发生了乙酯化反应，色谱保留行为得到明显改善，GLU/NAG和MPP的衍生产物分别在4.3 min和6.0 min出峰，且目标峰附近无干扰。以原甲酸三乙酯为衍生试剂，结果显示，在一级质谱图中观察不到理论衍生产物的目标峰，意味着衍生反应失败。因此，选择原乙酸三乙酯原液作为衍生化试剂。

同原乙酸三甲酯衍生化法，采用原乙酸三乙酯作为衍生化试剂，GLU和NAG会得到相同的衍生产物。由于出口茶叶需要检测GLU及其代谢物NAG、MPP的总和，因此没有必要增加实验步骤将GLU和NAG分开检测；且最新研究表明，GLU在茶叶中主要代谢为MPP^［28］^，意味着茶叶中NAG含量在GLU及代谢物总量中的占比很低，合并检测GLU和NAG对最终结果影响可忽略。综上，本研究在对实际样品中的GLU和NAG进行定量分析时，参考原乙酸三甲酯衍生化方法^［16］^。样品中的GLU和NAG经过提取和衍生步骤，均生成了GLU/NAG-TEOA，利用GLU的标准溶液建立工作曲线，对样品溶液中GLU/NAG-TEOA进行定量分析，从而实现GLU和NAG的合并检测，结果以GLU/NAG表示。

#### 2.3.2 衍生条件的优化

分析物与原乙酸三乙酯的酯交换反应需在酸性条件下进行，本实验比较了甲酸和乙酸两种挥发性酸对反应的影响。结果发现，使用甲酸时，衍生产物的响应仅为使用乙酸时的1/3，因此，选择乙酸作为衍生反应的催化剂。同时，实验发现标准溶液中的溶剂水可导致反应产率降低甚至反应失败，因此在衍生反应前，需用氮吹仪将溶剂吹干。

在分析物质量浓度为10 μg/L的条件下，优化了衍生温度。比较了在60、70、80、90和95 ℃反应后衍生产物的响应。结果见图3a，在60~95 ℃温度范围内反应后，目标化合物及同位素内标衍生产物均有较高响应，其中80 ℃时，响应最高，因此衍生温度选择80 ℃。

**图3:**
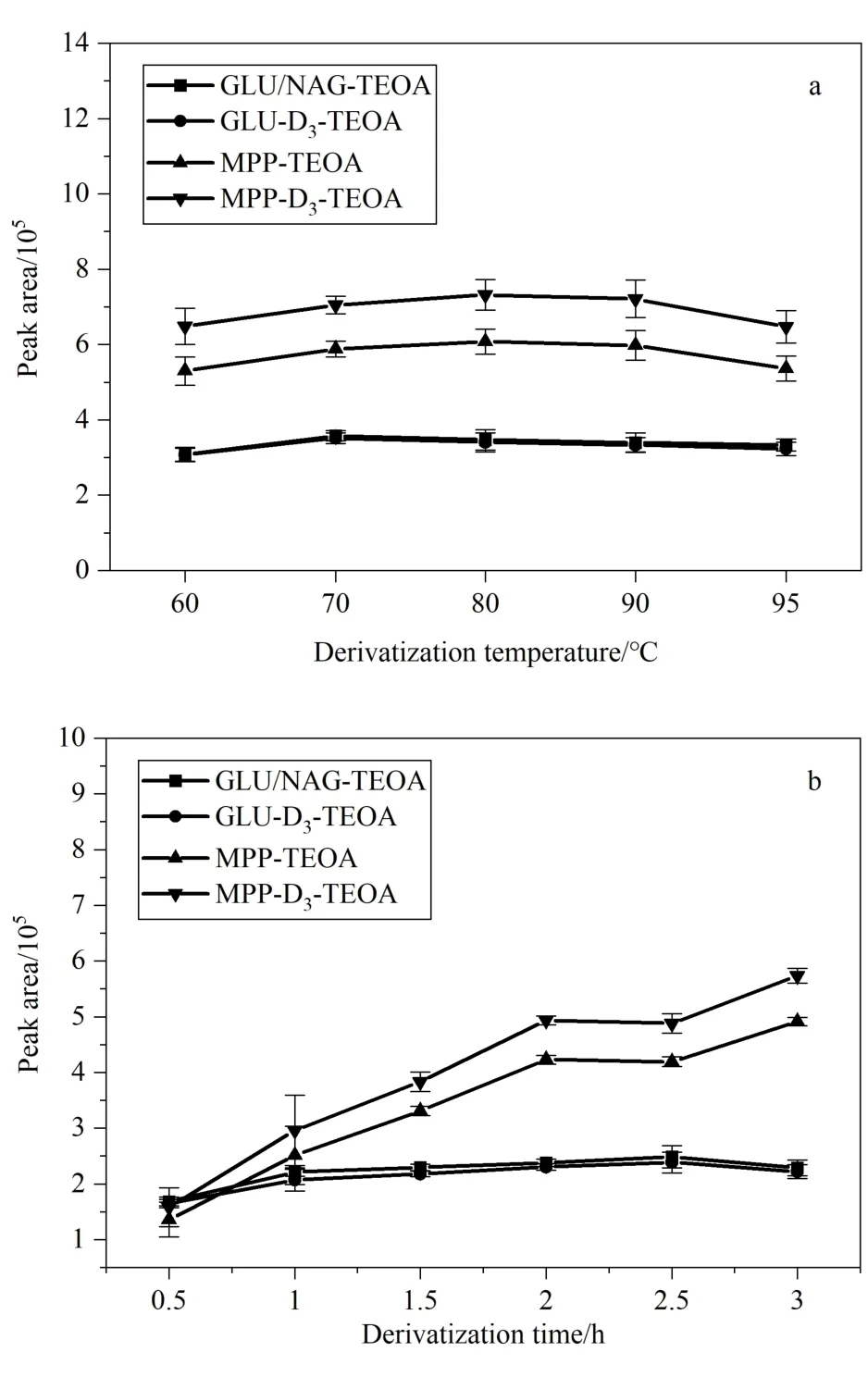
衍生（a）温度和（b）时间对衍生反应的影响（*n*=3）

实验进一步优化了衍生时间，衍生反应分别持续0.5、1、1.5、2、2.5和3 h，比较衍生产物的响应。结果见图3b，衍生反应1 h后，GLU/NAG-TEOA的峰面积基本趋于稳定；而MPP-TEOA的峰面积随着时间延长持续增大。虽然延长衍生时间有利于MPP获得更高的检测灵敏度，但同时也会影响实验的效率，切实可行的办法是在保证灵敏度的前提下，选择一个较短的衍生时间。通过分析数据，反应1.5 h时MPP-TEOA的响应较高且稳定性良好（峰面积RSD<5%），灵敏度满足要求，因此衍生反应时间选择1.5 h。综上，本实验以乙酸为催化剂，在80 ℃下反应1.5 h。

### 2.4 前处理条件的优化

草铵膦为强极性化合物，本研究以水作为提取溶剂，比较了超声5 min、涡旋1 min和振荡1 min 3种提取方式对回收率的影响。结果见图4，涡旋和振荡的提取回收率均介于99%~105%，而超声的提取回收率仅为50%，可能是由于超声方式不够剧烈，包埋在固体样品基质中的目标分析物未充分提取出来，因此本研究采用振荡的方法进行样品提取。

**图4:**
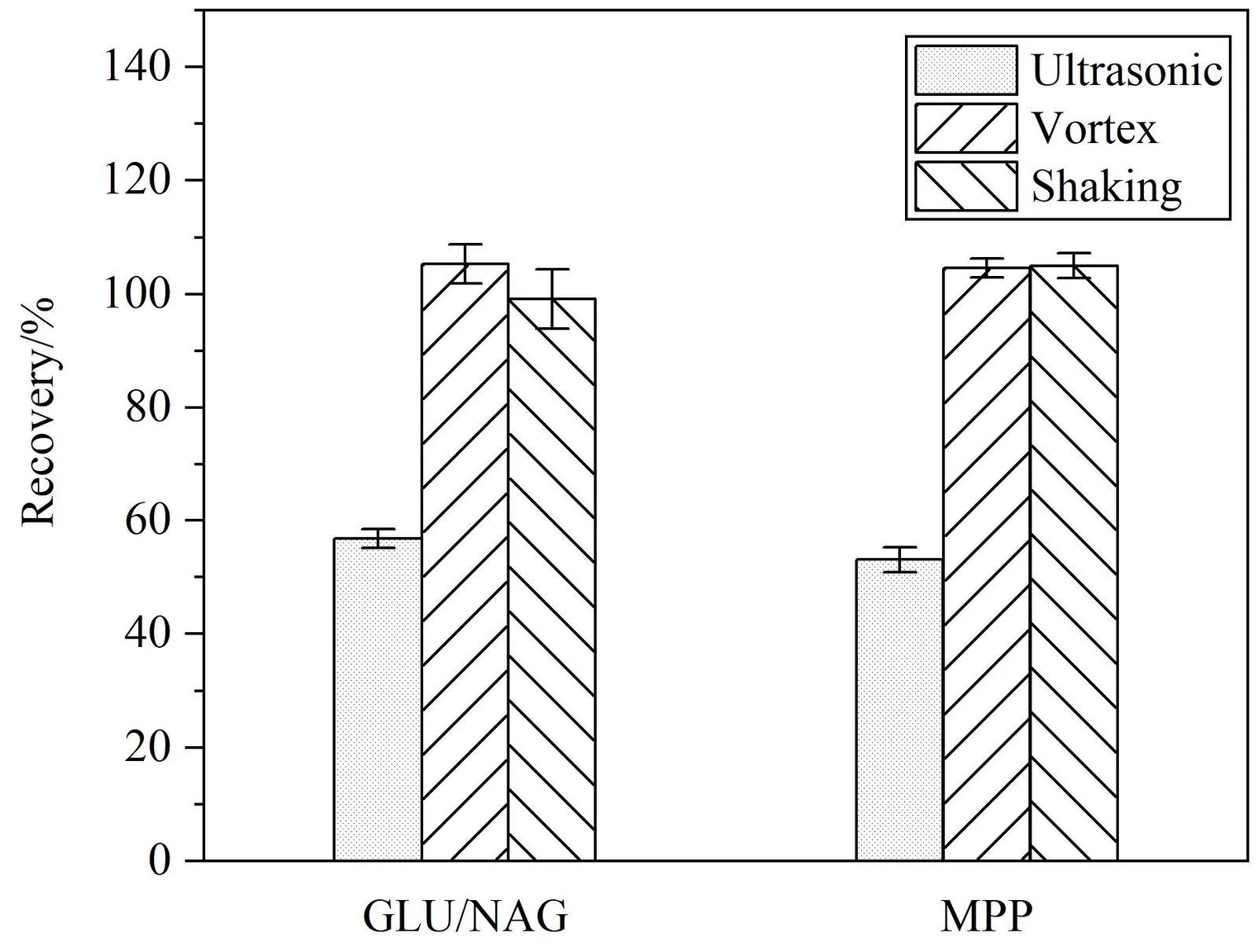
提取方式对绿茶中GLU/NAG和MPP回收率的影响（*n*=3）

草铵膦检测文献中常用方法的净化手段主要有HLB固相萃取柱法^［25，27，29，30］^、MWCNTs分散固相萃取法^［9，18，20，23］^以及不采用净化手段直接检测^［17］^。因此本研究比较了HLB固相萃取小柱（60 mg/3 mL）净化、MWCNTs分散固相萃取（10 mg）净化和不净化3种处理方式对回收率的影响。结果见图5，无论是否净化，化合物的回收率无显著差异。而且采用HLB固相萃取柱和MWCNTs分散固相萃取净化不仅成本更高，操作也更复杂。为节省成本，满足大批量样品快速检测的需要，因此前处理最终选择不采用固相萃取柱和分散固相萃取净化，提取溶液冷冻离心后直接进行氮吹和衍生步骤。

**图5:**
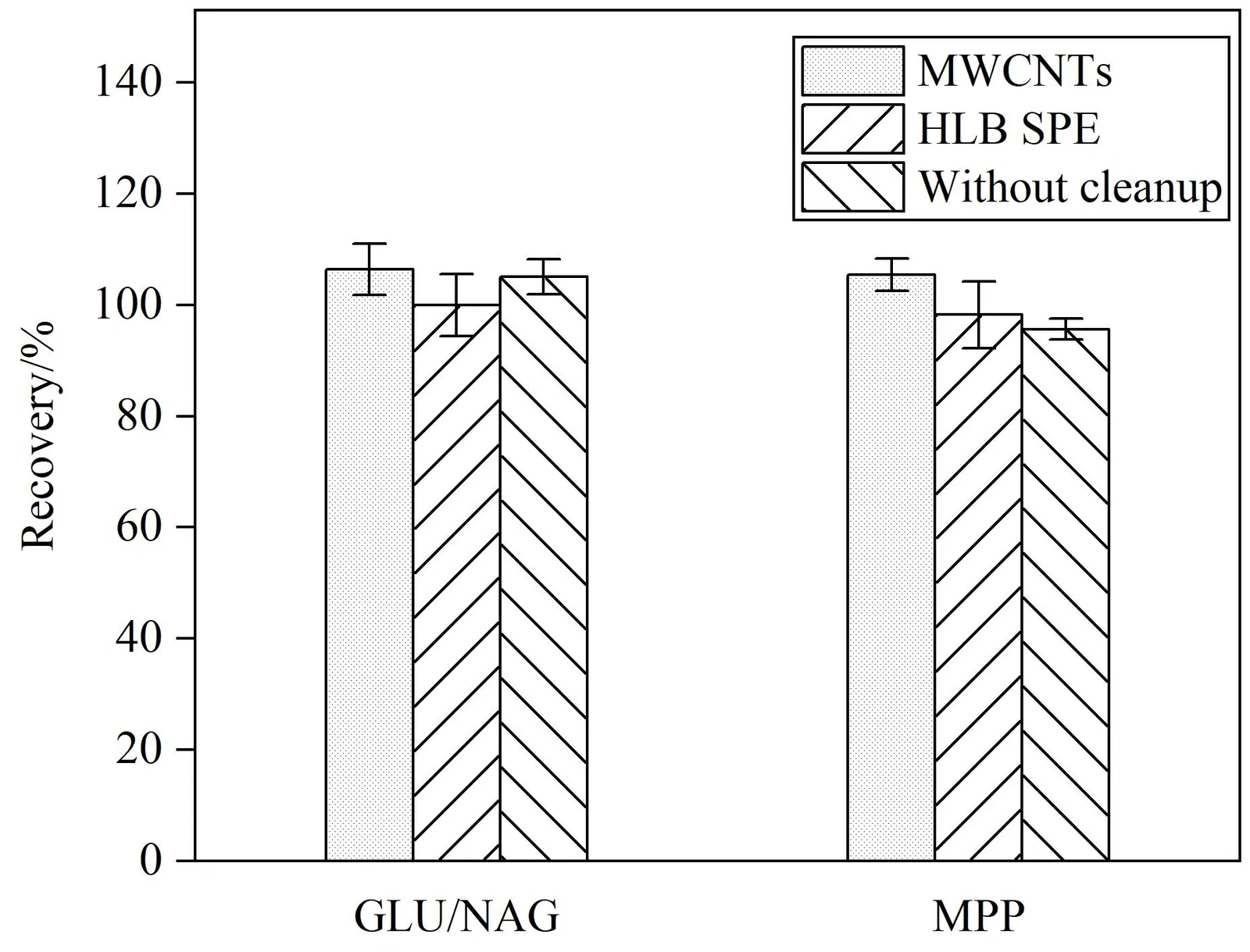
净化方式对绿茶中GLU/NAG和MPP回收率的影响（*n*=3）

### 2.5 线性范围、检出限和定量限

按1.2节方法制备系列校正标准工作液，并按1.3.2节仪器条件上机分析。以目标化合物与对应内标的质量浓度之比为横坐标（*x*），目标化合物与对应内标的定量离子峰面积之比为纵坐标（*y*），采用加权（权重系数为1/*x*）最小二乘法绘制标准曲线，并计算回归方程。在空白绿茶中进行加标，以3倍信噪比来确定方法的检出限；在满足信噪比≥10的前提下，将准确度和精密度均满足要求的最低浓度水平确定为方法的定量限。结果见表2，GLU/NAG和MPP在0.5~20 μg/L范围内的线性关系良好，相关系数*r*^2^>0.995，且校正标准品的反算浓度与实际浓度之间的相对偏差在‒20%~20%范围内^［31］^。GLU/NAG和MPP的检出限为2.2~5.3 µg/kg，定量限为25 µg/kg。

**表2:** GLU/NAG和MPP的线性方程、线性范围、相关系数、检出限和定量限

Compound	Linear equation	Linear range/（μg/L）	*r*^2^	LOD/（µg/kg）	LOQ/（µg/kg）
GLU/NAG	*y*=64.819171*x‒*0.165423	0.5*‒*20	0.99915	2.2	25
MPP	*y*=69.870442*x‒*0.008150	0.5*‒*20	0.99942	5.3	25

*y*： peak area ratio of analyte to internal standard； *x*： mass concentration ratio of analyte to internal standard.

### 2.6 基质效应

基质效应（ME）表示共流出组分对分析物电离效率的影响（基质增强或基质抑制），最终影响定量分析的准确性。通过以下公式计算ME值：ME=（基质匹配标准溶液的峰面积/溶剂标准溶液的峰面积‒1）×100%^［31］^。经计算，绿茶和红茶基质中GLU/NAG的ME值分别为‒42.1%和‒49.6%，MPP的ME值分别为40.0%和3.1%，表明在绿茶和红茶中两种化合物存在不同程度的基质抑制效应或增强效应。为消除基质效应，本研究采用稳定同位素标记内标法定量分析。

### 2.7 准确度与精密度

向绿茶和红茶基质空白样品中添加0.025、0.05、0.1和0.5 mg/kg 4个水平的标准溶液，每个水平重复5次。结果见表3，目标分析物的平均回收率为83.9%~105.0%，日内精密度（*n*=5）为1.9%~6.9%，日间精密度（*n*=10）为2.6%~10.3%。回收率和精密度结果均满足SANTE 11312/2021和农业部公告2386号规定的接受标准^［31，32］^。

**表3:** GLU/NAG和MPP的回收率和日内、日间精密度

Compound	Spiked level/（mg/kg）	Green tea			Black tea		
		Recovery/%	Intra-day RSD/% （*n*=5）	Inter-day RSD/% （*n*=10）	Recovery/%	Intra-day RSD/% （*n*=5）	Inter-day RSD/% （*n*=10）
GLU/NAG	0.025	102.9	2.9	5.9	105.0	2.9	6.3
	0.05	92.6	5.0	4.0	95.0	6.9	5.9
	0.1	89.2	4.1	3.4	83.9	5.5	5.2
	0.5	89.9	1.9	2.6	90.5	4.5	4.6
MPP	0.025	98.5	3.8	8.8	99.1	3.7	10.3
	0.05	86.9	5.0	5.6	89.6	4.8	5.7
	0.1	85.7	2.6	3.1	92.8	4.4	7.1
	0.5	87.6	3.5	5.5	88.5	2.7	5.2

### 2.8 实际样品分析

采用新开发的方法，测定了市售的34批样品（绿茶11批、红茶8批、乌龙茶7批、茉莉花茶4批、普洱茶2批、铁观音1批，白茶1批）。在9批次样品中检出GLU/NAG，其中4批次样品中同时检出MPP，阳性测试结果见表4。检出的GLU/NAG残留量低于我国最大残留限量，样品均为合格产品，但以欧盟的草铵膦最大残留限量判定，则有2批绿茶（序号2和3）为不合格产品。茶叶中草铵膦总体阳性检出率为26.5%，说明草铵膦在茶园中使用较多，其残留情况需要引起一定的关注。

**表4:** 实际样品中草铵膦及其代谢物的阳性测试结果

No.	Type of tea	Contents/（μg/kg）		
		GLU/NAG	MPP	Sum （expressed as GLU）
1	green tea	36	ND^*^	36
2	green tea	40	65	117
3	green tea	50	405	532
4	black tea	31	<25	31
5	black tea	60	<25	60
6	oolong	<25	ND	/
7	oolong	<25	ND	/
8	jasmine tea	<25	ND	/
9	white tea	<25	ND	/

Sum=content of GLU+content of MPP×molecular mass of GLU/molecular mass of MPP； ND： not detected.

## 3 结论

本研究建立了柱前衍生-超高效液相色谱-串联质谱法检测茶叶中草铵膦及其代谢物的定量分析方法。与GB 23200.122-2026相比，本方法的灵敏度更高。本方法的固定相采用普通C18色谱柱，流动相采用甲酸-甲酸铵缓冲盐溶液和乙腈，该色谱体系与大多数农药残留检测方法兼容，相对于上述国标方法，使用本方法则会减少实验操作过程中更换流动相和色谱柱的频率。该方法操作简便，成本低廉、灵敏度高，可完全满足出口茶叶中草铵膦的检测需求，具有较高的实用价值。

## References

[1] Yang X R， Sun W Q， Yang X， et al . China Tea Processing， 2025（1）： 63

[2] Yang Y J， Zhang B . World Pesticides， 2020， 42（3）： 20

[3] Gao W J， Zhang Y Z， Tong M M， et al . Journal of Tea Science， 2019， 39（5）： 587

[4] Xu H Z， Ren M X， Jiang Z Y， et al . Tropical Agricultural Engineering， 2021， 45（5）： 161

[5] Fu Q M， Wang D W， Luo Y Y， et al . Chinese Journal of Pesticide Science， 2024， 26（5）： 845

[6] Wu X G， Chen X Q， Xiao H J， et al . Chinese Journal of Chromatography， 2015， 33（10）： 1090 10.3724/sp.j.1123.2015.04045 26930967

[7] Ye M J， Lu X L， Liu X Z， et al . Chinese Journal of Chromatography， 2018， 36（9）： 873 10.3724/SP.J.1123.2018.04026 30251515

[8] Yang M， Sun S， Liu W F， et al . Food Science， 2019， 40（10）： 337

[9] Li Z Q， Yang M， Zhang X Z， et al . Journal of Tea Science， 2023， 43（2）： 263

[10] Liu W J， Lin J， Fu J W， et al . Chinese Agricultural Science Bulletin， 2020， 36（25）： 129

[11] Tsuji M， Akiyama Y， Yano M . Anal Sci， 1997， 13（2）： 283

[12] Tseng S H， Lo Y W， Chang P C， et al . J Agric Food Chem， 2004， 52（13）： 4057 10.1021/jf049973z 15212448

[13] Ohara T， Yoshimoto T， Natori Y， et al . Nagoya J Med Sci， 2021， 83（3）： 567 10.18999/nagjms.83.3.567 PMC8437997 34552290

[14] Rosales C A， Sheedy K L， Wasslena K V， et al . J Am Soc Mass Spectrom， 2024， 35（1）： 140 10.1021/jasms.3c00376 38127770

[15] Sasano R， Sekizawa J， Saito I， et al . Food Chem X， 2024， 24： 101806 10.1016/j.fochx.2024.101806 PMC11408379 39296482

[16] Joint Food and Agriculture Organization of the United Nations/World Health Organization Meeting on Pesticide Residues . Pesticide Residues in Food 2012 Evaluations. ［2025-07-11］. https://www.fao.org/4/i3111e/i3111e.pdf https://www.fao.org/4/i3111e/i3111e.pdf

[17] Ping H， Zhao F， Li C， et al . Chinese Journal of Chromatography， 2022， 40（3）： 273 10.3724/SP.J.1123.2021.08005 PMC9404154 35243837

[18] Zhang L J， Liu L J， Wang Y， et al . Food Science， 2023， 44（2）： 345

[19] Lattanzio V M T， Ciasca B， Verdini E， et al . Food Addit Contam A， 2023， 40（10）： 1345 10.1080/19440049.2023.2255302 37728620

[20] Zheng Y H， Yang Q H， Jiang W L， et al . Chinese Journal of Pesticide Science， 2024， 26（6）： 1161

[21] Jesús F， Rosa García A， Stecconi T， et al . Anal Bioanal Chem， 2024， 416： 675 10.1007/s00216-023-04946-7 37749278

[22] Rampazzo G， Gazzotti T， Pagliuca G， et al . LWT-Food Sci Technol， 2024， 200： 116159

[23] Wu Y， Zhou Y， Jiao X， et al . Anal Bioanal Chem， 2024， 416： 663 10.1007/s00216-023-04542-9 36693955

[24] Ji L Y， Wang P P， Zhu L Z， et al . Plant Protection， 2024， 50（2）： 235

[25] Yang X， Xie Z G， Li X L， et al . Chinese Journal of Chromatography， 2025， 43（2）： 155 10.3724/SP.J.1123.2024.03008 PMC11758234 39844706

[26] Eichhorn E， Vetter W， Anastassiades M . J Chromatogr A， 2025， 1762： 466327 10.1016/j.chroma.2025.466327 40934703

[27] Zhang X G， Wang M， Wang X . Journal of Food Safety & Quality， 2025， 16（17）： 54

[28] Yu H， Li D， Wu Y， et al . J Hazard Mater， 2024， 473： 134542 10.1016/j.jhazmat.2024.134542 38776809

[29] Lin L M， Zhang J Y， Xiao F， et al . Journal of Instrument Analysis， 2022， 41（11）： 1702

[30] Sun W S， Zhu J J， Zhong H H， et al . Physical Testing and Chemical Analysis （Part B： Chemical Analysis）， 2021， 57（4）： 322

[31] European Commission / EURL . Analytical Quality Control and Method Validation Procedures for Pesticide Residues Analysis in Food and Feed SANTE 11312/2021 v2. （2024-01-01） ［2025-07-11］. https://food.ec.europa.eu/system/files/2023-11/pesticides_mrl_guidelines_wrkdoc_2021-11312.pdf https://food.ec.europa.eu/system/files/2023-11/pesticides_mrl_guidelines_wrkdoc_2021-11312.pdf

[32] Ministry of Agriculture . No. 2386 Bulletin of the Ministry of Agriculture of the People’s Republic of China. （2016-04-11） ［2025-10-19］. https://www.moa.gov.cn/govpublic/ZZYGLS/201604/t20160413_5093548.htm https://www.moa.gov.cn/govpublic/ZZYGLS/201604/t20160413_5093548.htm

